# The Utilization of Carbonated Steel Slag as a Supplementary Cementitious Material in Cement

**DOI:** 10.3390/ma17184574

**Published:** 2024-09-18

**Authors:** Xinyue Liu, Pengfei Wu, Xiaoming Liu, Zengqi Zhang, Xianbin Ai

**Affiliations:** 1 State Key Laboratory of Advanced Metallurgy, University of Science and Technology Beijing, Beijing 100083, China; b20200134@xs.ustb.edu.cn (X.L.); 15232859538@163.com (P.W.); 2 School of Metallurgical and Ecological Engineering, University of Science and Technology Beijing, Beijing 100083, China; 3 Institute of Energy Research, Jiangxi Academy of Sciences, Nanchang 330096, China; axbjxas@163.com

**Keywords:** steel slag, accelerated carbonation, carbon capture and utilization, supplementary cementitious material, cement

## Abstract

Carbon emission reduction and steel slag (SS) treatment are challenges in the steel industry. The accelerated carbonation of SS and carbonated steel slag (CSS) as a supplementary cementitious material (SCM) in cement can achieve both large-scale utilization of SS and CO_2_ emission reduction, which is conducive to low-carbon sustainable development. This paper presents the utilization status of CSS. The accelerated carbonation route and its effects on the properties of CSS are described. The carbonation reaction of SS leads to a decrease in the average density, an increase in the specific surface area, a refinement of the pore structure, and the precipitation of different forms of calcium carbonate on the CSS surface. Carbonation can increase the specific surface area of CSS by about 24–80%. The literature review revealed that the CO_2_ uptake of CSS is 2–27 g/100 g SS. The effects of using CSS as an SCM in cement on the mechanical properties, workability, volume stability, durability, environmental performance, hydration kinetics, and microstructure of the materials are also analyzed and evaluated. Under certain conditions, CSS has a positive effect on cement hydration, which can improve the mechanical properties, workability, bulk stability, and sulfate resistance of SS cement mortar. Meanwhile, SS carbonation inhibits the leaching of heavy metal ions from the solid matrix. The application of CSS mainly focuses on material strength, with less attention being given to durability and environmental performance. The challenges and prospects for the large-scale utilization of CSS in the cement and concrete industry are described.

## 1. Introduction

The rapid development of the economy and production has led to the emission of large amounts of greenhouse gas, resulting in several environmental problems, the most obvious of which is global warming [1,2,3]. The global emission of greenhouse gasses is approximately 40 billion tons annually, and the trend is growing [4]. CO_2_, as the largest contributor, accounts for approximately 74% of all greenhouse gas emissions [4]. The main reasons for the rapid increase in CO_2_ concentration are human activities and the combustion of fossil fuels, which are mainly emitted by the power production, cement, iron and steel, metallurgy, and petrochemical industries [5,6,7]. The iron and steel industry is energy-intensive, and each ton of steel production emits 1.85 tons of CO_2_ [1,8]. The iron and steel industry contributes 6–8% of the total global CO_2_ emissions [9,10,11]. In China, the iron and steel industry is the third largest carbon emitter after the power and cement industries, accounting for 10% of domestic CO_2_ emissions [12].

**Figure 1:**
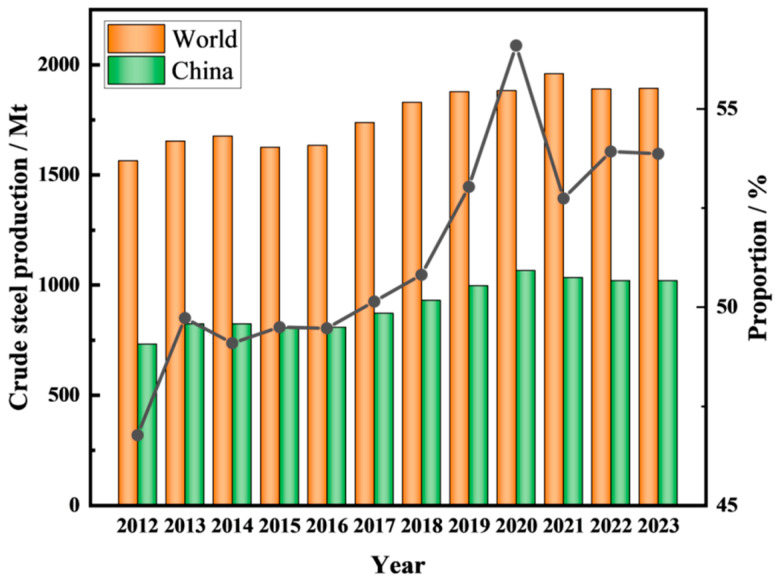
Crude steel production worldwide and in China from 2012 to 2023.

**Figure 2:**
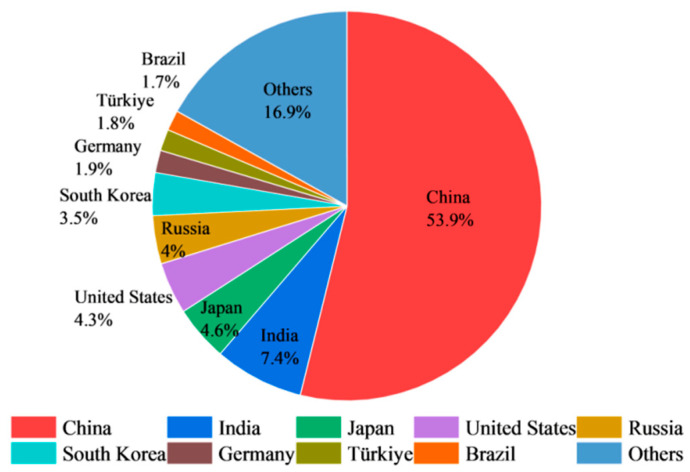
Crude steel production in different countries of the world in 2023.

The iron and steel industry is not only one of the largest contributors to carbon dioxide emissions but also one of the largest sources of industrial solid waste [13]. Steel slag (SS) is an industrial solid waste produced during the steelmaking process in the iron and steel industry [14,15,16]. According to the statistics of the World Steel Association, Figure 1 shows the annual crude steel production in the world and in China from 2012 to 2023 and the proportion of annual crude steel production in China compared to that in the world [17]. The world produced 1892.036 million tons of crude steel in 2023, and China produced 1019.08 million tons, accounting for approximately 54% of the total [18]. Figure 2 shows the share of crude steel production in different regions of the world in 2023. The top five regions in terms of global crude steel production are China, India, Japan, the United States, and Russia, accounting for 74.2% of the total [18]. SS generally accounts for 10–15% of crude steel production [19]. The generation of SS is increasing annually with the rapid growth of steel production. SS production in 2023 is estimated to be in the range of 190 million to 280 million tons. SS can be categorized into basic oxygen furnace slag (BOFS, which accounts for approximately 80% of the total amount of SS), electric arc furnace slag (EAFS), and ladle furnace slag (LFS) by different steelmaking procedures [10,20,21].

The United States, France, Germany, Japan, and other developed countries pay great attention to the comprehensive utilization of SS, and the SS utilization rate is close to 97% [20]. In these countries, SS is mainly used in road engineering, internal recycling in iron and steel enterprises, and construction materials [22,23,24]. However, the SS utilization rate in China is 29.5%, which is much lower than that in developed countries [25,26]. A low utilization rate leads to the continuous accumulation of SS. The cumulative stockpile of SS in China currently exceeds 100 million tons [27,28]. The vast majority of SS is stockpiled in open air, occupying valuable land resources [29]. Some toxic and harmful substances in SS are transferred to the soil and groundwater after a certain period of weathering and erosion, causing serious pollution in soil and water sources [30,31,32]. At the same time, the open storage of SS will also produce a large amount of dust, resulting in hazy weather and endangering human health. Therefore, the increase in SS reserves is an urgent problem for China [33].

Industrial solid wastes, such as SS, red mud, yellow phosphorus slag, and fly ash, have potential pozzolanic activity and can be used as substitutes for binders in cementitious materials [34,35,36,37,38,39]. Furthermore, replacing one ton of cement with solid waste can reduce carbon emissions by 700–850 kg. Consequently, SS-based cementitious materials promote low-carbon sustainable development. However, the hydration of f-CaO and f-MgO in SS causes the expansion and cracking of SS-based materials. Portlandite, f-CaO, f-MgO, and calcium silicate (C_2_S, C_3_S) in SS have good carbonation reactivity [21,40,41]. Therefore, SS is a good raw material for accelerated carbonation. The accelerated carbonation of SS refers to the reaction of calcium- and magnesium-containing minerals in SS with CO_2_ to form thermodynamically stable carbonates [42,43,44]. Carbonation treatment not only effectively reduces the content of f-CaO in SS, which is conducive to controlling volume expansion, but also improves the durability and mechanical properties of SS-based cementitious materials [21,45,46,47,48]. This provides a promising approach to optimize solid waste and reduce CO_2_ emissions in the steel industry.

Previous reviews have mostly focused on the reaction process of SS carbonation and the effects of various factors on the CO_2_ uptake of SS and have briefly introduced the application of SS after carbonation in cementitious materials, concrete, blocks, and aggregates [49,50,51]. However, there is no review on the physicochemical properties of carbonated steel slag (CSS) and the treatment and utilization of CSS in cement. This paper reviews the application of SS after carbonation. First, the different carbonation routes of SS are summarized. Then, the properties of CSS are reviewed. Next, the effects of CSS as an SCM in cement on the mechanical properties, workability, volume stability, durability, environmental performance, hydration kinetics, and microstructure of the materials are analyzed and evaluated. Finally, the challenges and prospects for the application of CSS in the cement and concrete industry are discussed.

## 2. Accelerated Carbonation of SS

**Figure 3:**
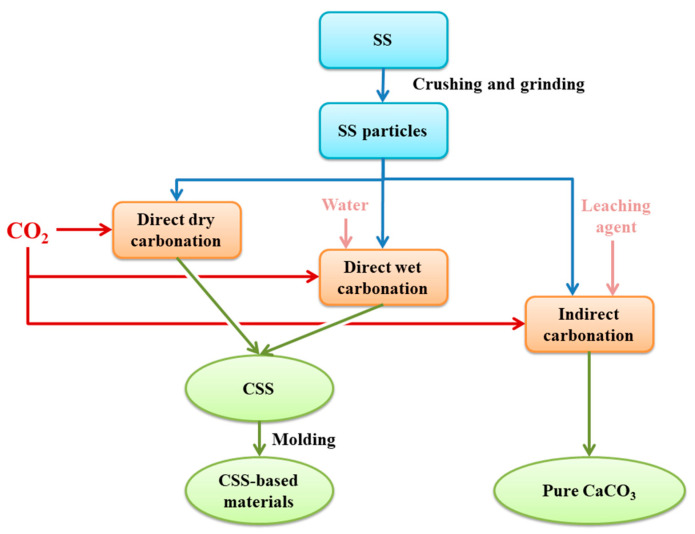
Scheme of accelerated carbonation of SS.

Accelerated carbonation, also known as mineralization, was first proposed by Seifritz in *Nature* in 1990 [52]. Mineralization mainly simulates the natural weathering process of minerals containing CaSiO_3_ and MgSiO_3_, and CO_2_ fixation is achieved through the mineralization of calcium- and magnesium-rich alkaline solid waste and natural minerals in the form of stable carbonate minerals [5,53]. Figure 3 shows the process diagram of the accelerated carbonation of SS. It includes direct carbonation and indirect carbonation. Direct carbonation can be classified into dry carbonation and wet carbonation depending on the participation of the water phase.

### 2.1. Direct Dry Carbonation

Direct dry carbonation is the direct reaction of solid SS and CO_2_ at high temperatures, similar to natural weathering [1]. It is a gas–solid reaction without a liquid phase. CO_2_ diffuses from the gas phase and then reacts with the active components in SS [54]. The reaction equations are shown as Equations (1)–(3).

CaO + CO_2_ → CaCO_3_ (1)

MgO + CO_2_ → MgCO_3_ (2)

Ca(OH)_2_ + CO_2_ → CaCO_3_ + H_2_O (3)

Due to the dense structure of SS, it is difficult for CO_2_ to diffuse to the inside of SS. The dense calcium carbonate generated by the carbonation process covers the surface of the SS and wraps the unreacted core, which further increases the difficulty of CO_2_ diffusion [55]. Therefore, direct dry carbonation under natural conditions is a slow process with poor carbonation efficiency. High-temperature, high-pressure, and grinding treatments are required to improve the mineralization efficiency of SS, but these treatments consume a large amount of heat and electricity [1,9,55,56].

However, the carbonation conversion rate was 50% under wet conditions when the liquid-to-solid ratio (L/S) was 0.4 L/kg at 50 °C and 3 bar pressure [57]. The main reason is that the calcium ions in SS easily react with CO_2_ when water is present, thus enhancing the carbonation efficiency. Therefore, researchers have added water to direct dry carbonation to promote carbonation conversion.

### 2.2. Direct Wet Carbonation

Direct wet carbonation refers to the reaction of SS directly with CO_2_ in a liquid phase. First, gas-phase CO_2_ diffuses into the liquid film on the SS surface and dissolves in the liquid phase to form H_2_CO_3_, which ionizes to H^+^, HCO_3_^−^, and CO_3_^2−^. Moreover, the calcium- and magnesium-containing components in SS dissolve and ionize Ca^2+^/Mg^2+^, which diffuses to the SS surface. Then, carbonate precipitation occurs [58,59,60,61]. The reaction equations are shown in Equations (4)–(8). Carbonation occurs both inside and on the surface of SS with the diffusion of CO_2_ and the dissolution of Ca^2+^. A dense layer of CaCO_3_ is formed on the SS surface, preventing the continuation of the carbonation reaction, which results in incomplete carbonation of the SS inside. The carbonation process also forms a silica layer, which further hinders the penetration of CO_2_ [62,63,64,65]. Therefore, the dissolution of calcium and magnesium ions and the diffusion of CO_2_ are the key factors affecting carbonation efficiency [5].

CO_2_ (g) + H_2_O (l) → H_2_CO_3_ (aq) → H^+^ (aq) + HCO_3_^−^ (aq) (4)

HCO_3_^−^ (aq) → H^+^ (aq) + CO_3_^2−^ (aq) (5)

Ca/Mg-silicates (s) + H^+^ (aq) → Ca^2+^/Mg^2+^ (aq) +SiO_2_ (s) + H_2_O (aq) (6)

Ca^2+^/Mg^2+^ (aq) + HCO_3_^−^ (aq) → (Ca/Mg)CO_3_ (s) + H^+^ (aq) (7)

Ca^2+^/Mg^2+^ (aq) + CO_3_^2−^ (aq) → (Ca/Mg)CO_3_ (s) (8)

Direct wet carbonation is similar to direct dry carbonation and is a one-step process. CSS is a mixture of carbonation products and raw SS that is difficult to separate and can be used as an SCM in cement and concrete.

### 2.3. Indirect Carbonation

Indirect carbonation has two steps: leaching and precipitation. The leaching process uses acids or other solvents to extract the reactive components (Ca^2+^ and Mg^2+^) from SS. Then, precipitation occurs during the reaction of the leaching solution with CO_2_ under alkaline conditions [66].

The SS leaching process is affected by a variety of factors, such as particle size, reaction temperature, reaction time, CO_2_ pressure, L/S, and other parameters [47]. The type and concentration of solvent are the main parameters impacting the indirect carbonation of SS. The different kinds of solvents used for the extraction of elements from SS vary greatly. Some leach impurities and some recover difficulty, causing new pollution problems; thus, the choice of solvent is particularly important. To date, the most commonly used leaching solvents are CH_3_COOH, HCl, HNO_3_, H_2_SO_4_, NH_4_C1, NH_4_NO_3_, and (NH_4_)_2_SO_4_ [54,67,68]. The carbonate precipitation process has a different pH range than SS leaching and requires high pH conditions; thus, indirect carbonation tends to maximize carbonation efficiency by adjusting the pH in steps (pH swinging) [5,69].

Indirect carbonation can be used to produce higher purity carbonate calcium due to the removal of impurities prior to carbonate precipitation [70], and the process has been commercialized. The world’s first indirect carbonation pilot plant was designed, constructed, and tested at Aalto University in Finland using SS as the raw material and NH_4_Cl as the leaching agent in 2014 [71]. The pilot plant produced approximately 10 kg of calcium carbonate with 99.5% purity using 190 L of liquid solvent and 20 kg of SS in batch mode. The process achieves nearly 80% calcium extraction and more than 70% carbon dioxide conversion, as well as regeneration of the NH_4_Cl solvent. This article focuses on the utilization of SS after accelerated carbonation in cement and concrete and does not consider the process of indirect carbonation.

## 3. Physicochemical Properties of CSS

The physicochemical properties of the CSS are shown in Table 1 and are described in more detail in Section 3.1 (for mineral composition), Section 3.2 (for physical properties), and Section 3.3 (for CO_2_ uptake).

### 3.1. Mineral Composition

The mineral phase of CSS is closely related to the type of SS minerals and the carbonation conditions [9]. SS minerals have a greater influence than carbonation conditions, as shown in Table 2. Carbonation activity is determined by the amount of carbonated reactive calcium, not the content of CaO in the SS [82,83]. Calcium in the form of f-CaO and portlandite has the highest susceptibility to carbonation [75,84]. The carbonation activities of C_2_S and C_3_S are the next highest. γ-C_2_S has greater carbonation reactivity than β-C_2_S [85]. Calcium oxide minerals structurally bound to elements (Mg, Fe or Al) exhibit low carbonation reactivity [83]. Other minor oxides, such as FeO, calcium iron oxide, and magnbesioferrite, do not participate in the carbonation reaction [75,80].

After carbonation, the peaks of easily carbonated f-CaO and Ca(OH)_2_ basically disappear [72,86]. The intensity of the γ-C_2_S and β-C_2_S peaks decreases, and the peak intensity of CaCO_3_ subsequently increases [80,82,85]. The carbonation products are near-amorphous C-S-H and calcium carbonate, the main form of which is calcite with traces of aragonite and magnesite [85]. Magnesium may be incorporated in calcite [84]. The carbonation products of γ-C_2_S, β-C_2_S, and C_3_S are different. γ-C_2_S and C_3_S generatemainly calcite, together with aragonite and vaterite [85,87]. β-C_2_S produces mainly aragonite, but the formation of calcite, vaterite, and amorphous silica-rich phases during β-C_2_S carbonation has also been studied [88]. Additionally, C_2_S and C_3_S produce amorphous calcium silicate hydrocarbonate.

**Figure 4:**
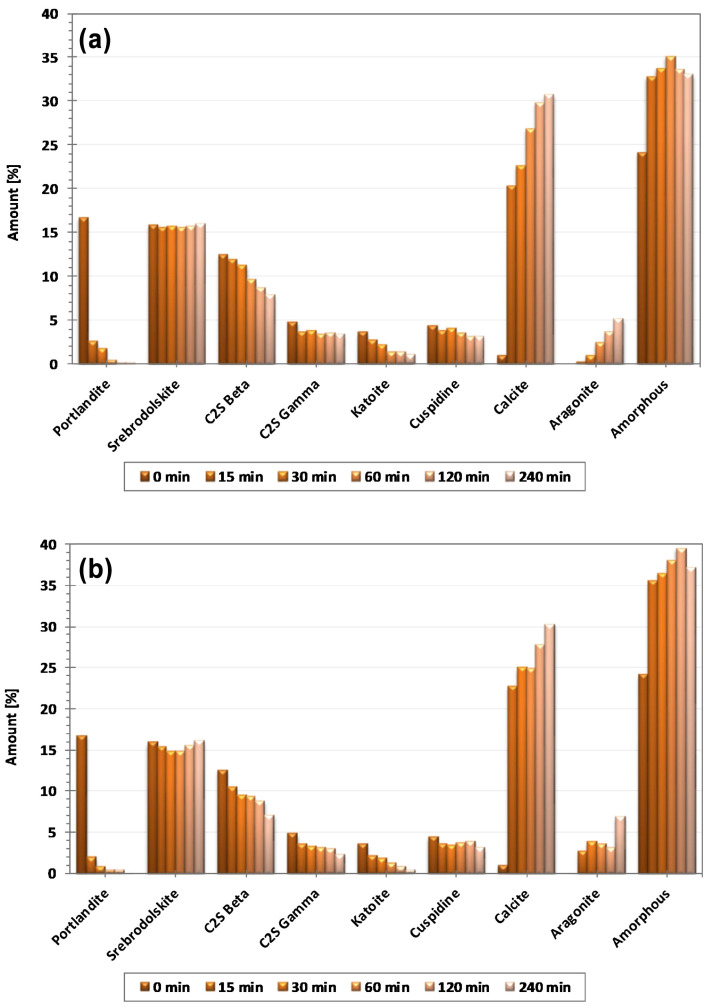
XRD results obtained for carbonated BOF slags at 1.3 bar (**a**) and 10 bar (**b**) CO_2_ pressure [89].

Figure 4 shows the mineral phase composition of BOFS at different times. The carbonation reaction is very rapid during the first 15 min due to the transformation of portlandite to calcite, amorphous calcium carbonate, and aragonite [89]. The slower-reacting calcium silicate phases in SS continue to react and produce more calcite as the carbonation time increases [73].

### 3.2. Physical Properties

Accelerated carbonation has four main effects on the physical properties of SS: (1) a decrease in the average density, (2) an increase in the specific surface area, (3) a refinement of the pore structure, and (4) a change in the surface morphology/particle shape [77,90].

**Figure 5:**
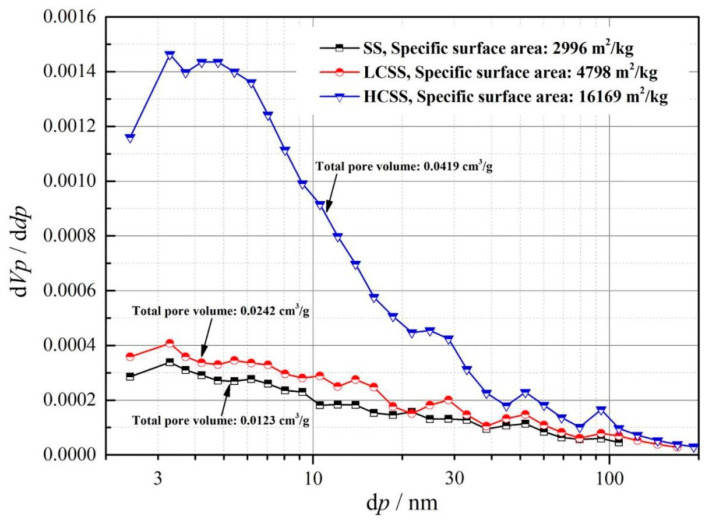
Pore structures and specific surface areas of SS, LCSS, and HCSS (BET) [73].

After carbonation by a rotating packed bed reactor at 30 °C for 3 h, the specific gravity of the BOFS reduced from 3.13 g/cm^3^ to 2.66 g/cm^3^, and the Blaine specific surface area increased from 672 m^2^/kg to 1020 m^2^/kg, which was mainly due to the formation of fine carbonates [78]. Carbonation increased the specific surface area of the EAFS from 1.7 m^2^/g to 13.9 m^2^/g [79]. The carbonation of SS for 15 min resulted in a low CO_2_ uptake of 4.6% (LCSS), and the carbonation of SS for 240 min resulted in a high CO_2_ uptake of 13.2% (HCSS). The pore size distributions of SS, LCSS, and HCSS are shown in Figure 5. The specific surface area of CSS after carbonation for 15 min and 240 min increased from 2996 m^2^/kg to 4798 m^2^/kg and 16,169 m^2^/kg, respectively [73]. The number of pores less than 30 nm in size increased in CSS. The higher the CO_2_ uptake, the larger the total pore volume. The calcium in the mineral phase of SS continuously dissolved, which destroyed the original dense microstructure of SS and produced more porous and fluffy carbonation products with a greater specific surface area during the carbonation process [74].

Both the particle size and density of BOFS decreased after carbonation [76]. This was caused by the gradual leaching of reactive calcium species from fresh BOFS, resulting in a less dense and shrinking matrix. The average particle size of the BOFS decreased from 8.73 μm to 6.99 μm when the fresh BOFS reacted with CO_2_, and the carbonation conversion was 17%. The mean particle size decreased to 5.23 μm when the carbonation conversion of BOFS increased to 48%.

**Figure 6:**
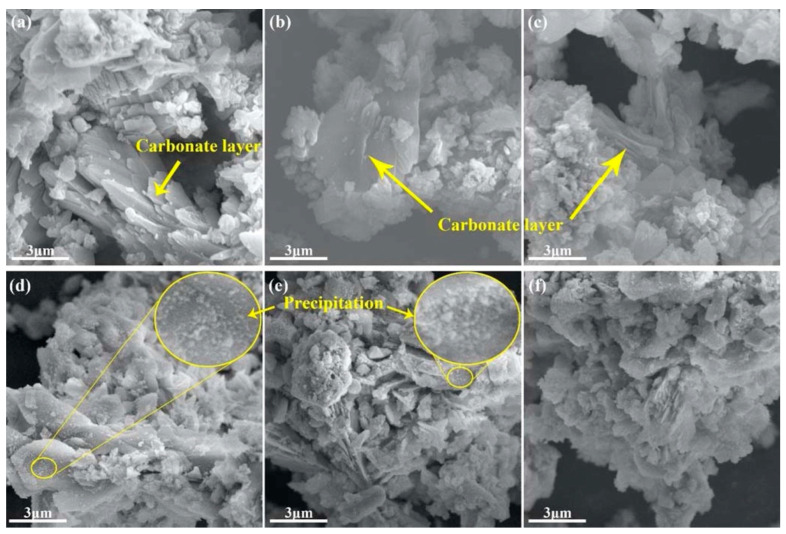
Microstructure of CSS: (**a**–**c**) the carbonate-layered carbonation products on the outer surface of the SS particles; (**d**–**f**) unevenly deposited calcite particles on the surface of the SS particles [80].

Uncarbonated SS shows a smooth and dense surface without crystalline precipitates [72]. After carbonation, rhombic or cubic calcite crystals 0.5–3 μm in size attach to the surface of CSS, and needle-like aragonite is also observed [76,77,79,81]. The microstructure of CSS is shown in Figure 6. The main carbonation products have two forms on the cracks and pore surfaces of SS [80]. One part of the carbonate-layered carbonation products precipitates on the outer surface of the SS particles, as shown in Figure 6a–c. The other calcite particles are deposited unevenly on the surface of the SS particles, as shown in Figure 6d–f.

**Figure 7:**
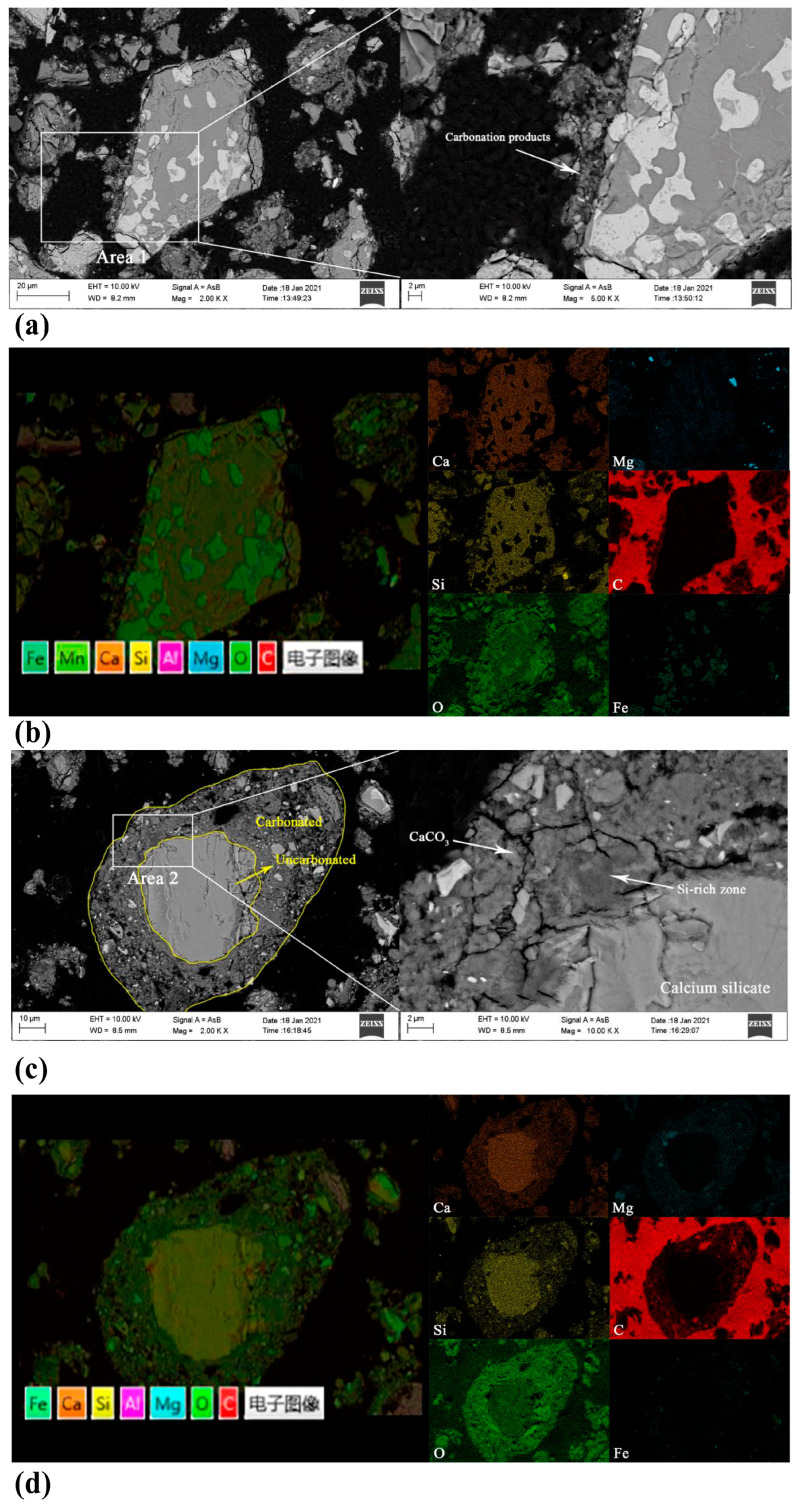
BSEM images: (**a**) LCSS; (**b**) element mapping of LCSS; (**c**) HCSS; and (**d**) element mapping of HCSS [73].

**Figure 8:**
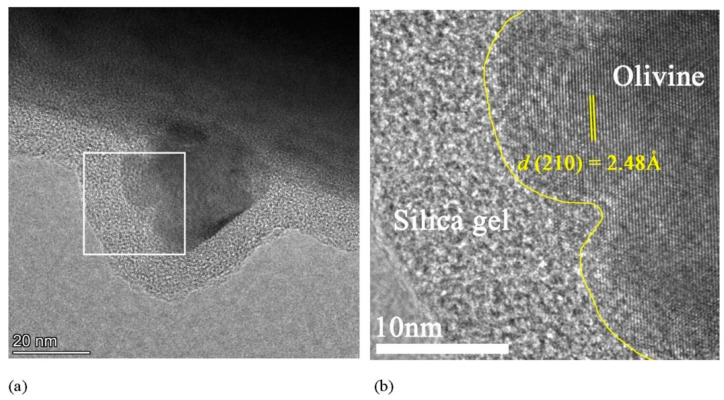
High-resolution transmission electron microscopy images of (**a**) CSS and (**b**) CSS enlarged from [74].

The original structure of the SS particles almost completely disappeared with a prolonged carbonation time, and more carbonation products grew on the SS surface, forming a core–shell structure within the CSS particles [73]. Figure 7 shows typical BSEM images of LCSS and HCSS. A porous and rough product layer with a thickness of 5–10 μm formed around the SS particles, and the product layer composed of CaCO_3_ and amorphous SiO_2_ gel consisted of a large number of needle-like carbonation products with a diameter of 10–20 nm [74]. Only a small portion of the core consisting of calcium silicate and iron-containing phases was uncarbonated [73]. A high-resolution transmission electron microscopy image of the CSS is shown in Figure 8, which shows the core–shell structure of the CSS [74].

### 3.3. CO_2_ Uptake

The CO_2_ uptake of CSS is related to the carbonation time, temperature, CO_2_ concentration, L/S, gas flow rate, and particle size [81,91,92,93,94]. Table 3 shows the CO_2_ uptake of direct carbonation under different conditions. Most of the carbonation reactions occur in the first hour. CO_2_ uptake decreases significantly after 4 h [81]. The reaction temperature affects system parameters such as CO_2_ dissolution, calcium leaching, reaction kinetics, and equilibrium simultaneously. The rate of carbonation increases significantly with an increasing reaction temperature. Generally, CO_2_ uptake at an L/S of 10 is good. This is because the concentration of reactive substances in the slurry is greater at a low L/S than at a high L/S, resulting in a greater chemical potential driving force for the carbonation reaction. CO_2_ uptake increases when the stirring speed reaches 300–500 rpm [76]. The resistance to mass transfer (i.e., liquid film thickness) decreases with the increase in the stirring speed in this range. However, CO_2_ uptake decreases when the stirring speed is further increased, showing that the reduction in mass transfer resistance is compensated by the decrease in retention time at higher stirring speeds.

## 4. The Application of CSS as an SCM in Cement

The accelerated carbonation of SS not only reduces the contents of f-MgO and f-CaO in SS but also solves the stability problem of SS-based cementitious materials [95]. In addition, the CaCO_3_ particles produced by carbonation can be used as the nucleus for silicate hydration, which leads to the formation of more hydrated calcium silicate in the system and improves the early strength of cementitious materials [96]. Thus, CSS can be used as an SCM in cement and concrete [21]. This not only enables the complete resource utilization of SS but also significantly reduces carbon emissions.

### 4.1. The Test Methods Used for CSS Cement

The mechanical properties of CSS cement were tested according to the GB/T 20491–2017 standard “Steel slag powder used for cement and concrete” [73,74,97]. CSS was mixed with ordinary Portland cement (OPC) at a 30 wt% cement replacement ratio to prepare 40 mm × 40 mm × 40 mm mortar with a water-to-binder ratio of 0.5 and a binder-to-sand ratio of 1:3. The mortar samples were demolded after 1 d and then cured in water at 20 ± 2 °C. The compressive strengths of mortars at different ages of 3 d, 7 d, 28 d, and 90 d were tested by using a mechanical test system with the GB/T 17671–2021 standard “Test method of cement mortar strength (ISO method)” [98]. The average compressive strength of six cement pastes was recorded for each test. In addition, other water-to-binder ratios, binder-to-sand ratios, and curing methods were used in the preparation of the samples, as shown in Table 4.

The workability of cement pastes with different CSS dosages was measured using a miniature slump cone mold with a height of 60 mm, a top diameter of 36 mm, and a bottom diameter of 60 mm [99]. The average diameter of the fresh mixture expanding in four directions was measured after 15 jolting cycles. The water demand and setting time were tested using a Vicat needle according to GB/T 1346–2011 “Test methods for water requirement of normal consistency, setting time and soundness of the portland cement” [100].

The volume stability of CSS cement pastes was measured using an autoclave test [74]. Prismatic cement pastes with a size of 25 mm × 25 mm × 280 mm were cured for 1 d at 20 ± 2 °C and a relative humidity of more than 95%. Then, the paste specimens were demolded, and the initial lengths were immediately measured and designated as L_0_. The specimens were autoclaved at 216 °C and 2.0 MPa for 6 h in an autoclave chamber. Then, the pastes were taken out after the pressure decreased to a normal atmospheric pressure and slowly cooled to room temperature in water. The lengths of the paste specimens were measured again when their temperatures declined to room temperature and designated as L_1_. The autoclave expansion of the paste specimen was calculated according to Equation (9), where L_A_ is the autoclave expansion (%) and L is the effective length of the paste specimen, i.e., 250 mm. Additionally, the soundness can also be determined using the Le Chatelier expansion test according to GB/T1346–2011 [99,100].

L_A_ = (L_1_ − L_0_) × 100/L (9)

The durability of sulfate expansion of mortars was determined according to ASTM C1012 [78,101]. The specimens were cured in lime-saturated water until the age of 28 d and then immersed in a 5% Na_2_SO_4_ solution at 23 ± 1 °C. The length change was monitored for 6 months. The toxicity characteristic leaching procedure for CSS was carried out in accordance with NIEA R201.14C [76]. The hydration heat of CSS pastes was monitored using an isothermal calorimeter at 20 °C for 72 h [99]. The mineralogy of hardened pastes was analyzed with the XRD technique after stopping hydration using alcohol and crushing to obtain particles smaller than 45 mm [78].

### 4.2. The Mechanical Properties of CSS Cement

The CSS properties, carbonation conditions, and dosage all affect compressive strength development [21,102,103,104,105]. Table 4 shows the effects of different conditions on the strength of the CSS mortar and paste. The addition of SS greatly reduces the mechanical properties of cement mortar due to poor hydration reactivity [73]. Carbonation treatment increases the compressive strength of SS cement mortar and paste [76]. For example, the compressive strengths of cement pastes with 30% CSS are 91.0 MPa, 113.6 MPa, and 125.3 MPa at 7 d, 28 d, and 90 d, respectively, which are 11.5%, 9.7%, and 15.4% higher than those of OPC pastes with 30% SS [74].

Generally, the higher compressive strength of CSS can be attributed to both physical and chemical effects [103]. From a physical perspective, carbonation destroys the original structure of SS particles and forms a rough and porous surface with the generation of calcite and silica gel [76,106]. This not only provides more sites for the growth and nucleation of cement hydrates but also acts as a nanofiller in the gaps between cement particles, which leads to a denser structure at an early stage. In terms of chemical properties, CaCO_3_ reacts with C_3_A in cement to form monocarbonaluminate, which is larger and potentially harder than sulfoaluminate, increasing the bond strength between the CSS particles and the cement matrix [73,99,107]. The formula is shown in Equation (10). In addition, silica gel reacts directly with Ca(OH)_2_ to form C-S-H, which optimizes the pore structure of the cement matrix and facilitates the increase in compressive strength. Due to the synergistic effect of CaCO_3_ and SiO_2_ gel, the compressive strength of CSS mortar or paste is better than that of SS mortar or paste.

2C_3_A + 1.5CaCO_3_ + 0.5Ca(OH)_2_ + 22.5H_2_O → C_3_A·CaCO_3_·11H_2_O + C_3_A·5CaCO_3_·0.5Ca(OH)_2_·11.5H_2_O (10)

The compressive strength of the CSS mortar first increases and then decreases with increasing CO_2_ uptake [102,103,104]. Appropriate CO_2_ uptake can improve the hydration activity of SS, and excessive carbonation reduces the hydration activity [108]. This is due to the excessive consumption of calcium silicate with a prolonged carbonation time [76]. Moreover, the positive effect of the nucleation site disappears with an increase in the number of calcite particles. More even carbonation products form a thicker carbonation layer on the particle surface, which restricts the reaction between the CSS and cement matrix and ultimately reduces the hydration activity of the CSS. Therefore, the incorporation of CSS with a high CO_2_ uptake will adversely affect cement hydration.

**Table 4:** The effect of CSS on the compressive strength of the CSS mortar and paste.

SS	Carbonation Time (h)	CO_2_ Uptake (g/100 g SS)	Sample (Type)	Molding Condition			Curing Method	Compressive Strength (MPa)				Reference
				Mixture Composition	Water/Powder Ratio	Sand/Powder Ratio		3 d	7 d	28 d	90 d	
SS	0	0.7	40 × 40 × 40 mm (mortar)	100% OPC	0.5	3	cured in water at 20 ± 2 °C	35.1	44.5	55.2	60.2	[73]
				30% SS + 70% OPC				24.3	33.8	40.2	47.1	
	0.25	4.6		30% CSS + 70% OPC				24.9	35.6	43.4	50.3	
	4	13.2		30% CSS + 70% OPC				21.6	34.5	46.1	51.1	
SS	0	0.7	20 × 20 × 20 mm (paste)	100% OPC	0.3	-	cured in water at 20 ± 2 °C	96.9	108.1	114.9	135.3	[73]
				30% SS + 70% OPC				64.2	71.3	89.0	108.3	
	0.25	4.6		30% CSS + 70% OPC				62.3	78.5	95.1	116.4	
	4	13.2		30% CSS + 70% OPC				53.2	85.6	110.8	129.4	
SS	0	-	20 × 20 × 20 mm (paste)	100% OPC	0.28	-	cured in water at 20 ± 2 °C	101.0	113.0	126.1	143.9	[74]
				30% SS + 70% OPC				61.5	81.6	103.6	108.6	
	30	6.14		30% CSS + 70% OPC				60.6	91.0	113.6	125.3	
SS	0	-	40 × 40 × 40 mm (mortar)	100% OPC	0.5	3	cured in water at 20 ± 2 °C	36.2	47.4	57.0	68.6	[74]
				15% SS + 85% OPC				30.2	39.4	47.8	60.0	
				30% SS + 70% OPC				23.6	32.5	38.9	51.2	
	30	6.14		15% CSS + 85% OPC				30.9	42.6	49.9	62.8	
				30% CSS + 70% OPC				24.3	35.0	42.2	54.1	
BOFS	-	2.03	50 × 50 × 50 mm (mortar)	100% OPC	0.485	2.75	cured in lime-saturated water at 23 ± 1 °C	21.80 ± 0.67	26.07 ± 0.89	40.06 ± 1.02	-	[78]
				10% SS + 90% OPC				17.50 ± 0.34	21.34 ± 1.51	33.99 ± 1.53	-	
	-	20.3		10% CSS + 90% OPC				22.84 ± 0.55	26.51 ± 0.97	39.34 ± 1.06	-	
EAFS	-	-	30 × 30 × 30 mm (paste)	80% SS + 20% cement	0.4	-	cured in a chamber at 20 ± 2 °C and ≥ 90% for RH	-	-	17.1	-	[109]
	-	-		80% CSS + 20% cement				-	-	21.5	-	
LFS	0	-	50 × 50 × 50 mm (mortar)	100% OPC	-	2.75	cured in lime water	20.10 ± 2.21	28.69 ± 4.49	37.38 ± 1.63		[110]
				5% SS + 95% OPC				25.71 ± 2.34	30.03 ± 1.79	33.69 ± 1.21		
				10% SS + 90% OPC				23.17 ± 1.17	26.43 ± 2.45	33.09 ± 3.21		
				15% SS + 85% OPC				17.83 ± 4.81	26.27 ± 1.33	32.25 ± 1.99		
				20% SS + 80% OPC				11.30 ± 1.23	23.22 ± 2.44	25.76 ± 2.06		
	60	-		5% CSS + 95% OPC				29.32 ± 1.98	33.19 ± 0.92	38.65 ± 4.06		
				10% CSS + 90% OPC				24.34 ± 1.18	28.17 ± 2.29	37.94 ± 5.11		
				15% CSS + 85% OPC				21.61 ± 0.90	28.17 ± 2.29	33.05 ± 0.98		
				20% CSS + 80% OPC				20.48 ± 1.22	25.22 ± 1.56	31.34 ± 4.43		

Although a CSS with a high CO_2_ uptake reduces the early compressive strength of the paste, it enables the rapid development of the later compressive strength [73]. The pozzolanic activity of silica gel formed after carbonation may play a major role. The higher CO_2_ uptake of CSS produces more silica gels, generating more C-S-H during hydration. This results in a denser microstructure with smaller pore sizes and an enhanced interface between the CSS particles and the matrix, which contributes to the enhancement in the later compressive strength of the CSS paste.

The carbonation of SS can improve the mechanical properties of SS cement mortar. The excessive CO_2_ uptake of CSS may consume calcium silicate via hydration reactions, such as β-C_2_S and C_3_S, which adversely affects the early hydration activity of CSS and decreases the early strength of cementitious materials [103]. The balance between CO_2_ uptake and hydration activity needs to be considered to optimize the application of CSS in cementitious materials.

### 4.3. Workability of CSS Cement

The fresh properties and setting times of the cement pastes with different substitution ratios of SS and CSS are shown in Table 5. Adding large-particle-size CSS (e.g., CS1) improves workability, while small-particle-size CSS decreases workability compared to OPC paste [99]. This is partially because the reduction in the average particle diameter of the CSS expands the equivalent wetting surface of the particles in the fresh mix. A higher CO_2_ uptake of the small-particle-size CSS causes more rough, porous structures on the particle surface, which leads to higher yield stresses and viscosities and, subsequently, lower workability. The water demand for CSS cement paste increases with increasing dosage and CO_2_ uptake [110]. The high CO_2_ uptake of CSS with a large specific surface area enhances the water adsorption of the particles. The setting time of the CSS paste shortens as the CSS dosage increases compared to that of OPC. This is because calcium carbonate can provide additional nucleation sites for cement hydration, thus accelerating the hydration reaction [78]. However, the setting time of the CSS paste will be prolonged as the particle size of the CSS decreases and the CO_2_ uptake increases. The incorporation of CSS in cement requires comprehensive consideration of the effects of particle size, dosage, and CO_2_ uptake on the workability of CSS cement.

### 4.4. The Volume Stability of CSS Cement

Free-CaO and Ca(OH)_2_ in BOFS were eliminated after carbonation [77,78]. The extractable CaO content of the carbonated LFS decreased from 6.5% to 0.2–0.3% compared to that of the uncarbonated LFS [111]. Accelerated carbonation significantly reduces the f-MgO and f-CaO contents in CSS, thus improving the volume stability. The cement paste containing 30% CSS did not show excessive expansion or microcracking after a 6 h autoclave test. In contrast, the cement paste containing 30% uncarbonated SS did not pass the autoclave test and produced numerous cracks [74]. The volume expansion of CSS mortar significantly decreased compared with that of SS mortar, and the volume change in CSS mortar was stable with an increasing carbonation time [80,112]. The autoclave expansion of the uncarbonated SS specimen reached 1.9%, and that of CSS after 6 min of carbonation decreased to 0.48%, which enabled the safe use of SS [113].

**Figure 9:**
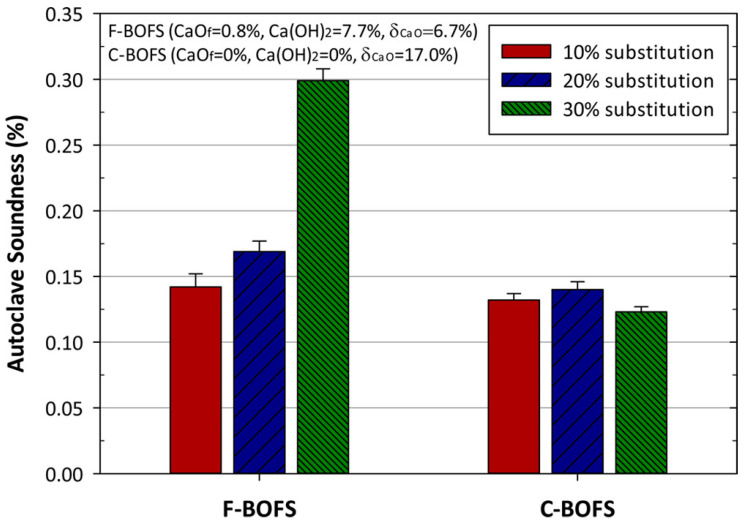
Autoclave soundness expansion of cement mortar with different substitution ratios using fresh BOFS (F-BOFS) or carbonated BOFS (C-BOFS) with carbonation conversion of 17% [76].

The autoclave expansion of cement mortars with different amounts of fresh BOFS (F-BOFS) or carbonated BOFS (C-BOFS) is shown in Figure 9. The f-CaO content of the mortar increased with increasing F-BOFS substitution, resulting in a greater expansion capacity of the mortar [76]. The maximum expansion of the mortar was approximately 0.3% at 20% F-BOFS doping. However, autoclave expansion was less than 0.15%, even at up to 30% C-BOFS doping. This was due to the conversion of reactive f-CaO in BOFS to relatively stable calcium carbonate by carbonation.

The volume stability of the CSS mortar is positively correlated with CO_2_ uptake, but a high CO_2_ uptake of the CSS may lead to low early hydration activity. Therefore, it is necessary to adjust the carbonation conditions to obtain a balance between volume stability and hydration activity.

### 4.5. The Durability of CSS Cement

**Figure 10:**
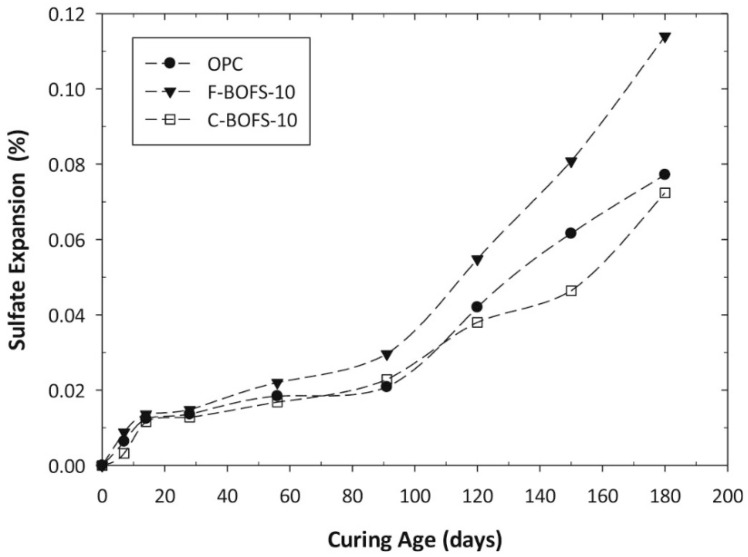
Sulfate expansion of cement mortar using 10% F-BOFS (carbonation conversion of 6%) or C-BOFS (carbonation conversion of 60%) immersed in 5% Na_2_SO_4_ [78].

The CSS cement mortar has good sulfate resistance compared with the SS cement mortar. The sulfate expansion resistance of the mortars immersed in 5% Na_2_SO_4_ solution is shown in Figure 10. The sulfate expansion of the F-BOFS-10 mortar is significant compared to that of the OPC control [78]. This may be due to the formation of expansive ettringite and gypsum from leachable calcium hydroxide in fresh BOFS. In contrast, the C-BOFS-10 mortar has a relatively low sulfate expansion relative to that of the F-BOFS-10 mortar, which meets the requirements of the American Concrete Institute 318 for anti-expansion limits (<0.10% at 6 months). CSS reduces the calcium hydroxide content and permeability, thereby limiting the supply of sulfate ions from sources outside the cement matrix.

### 4.6. Environmental Performance of CSS Cement

The accelerated carbonation of SS can significantly reduce the leaching concentration of heavy metals such as Ag, Cr, Cr(VI), Hg, and Ba from the solid matrix because a calcium carbonate layer forms on the CSS surface [76]. When the carbonation conversion increased to 48%, the leaching concentrations of Cr, Cr(VI), and Hg in BOFS were undetected, and the leaching concentration of total Cr was effectively reduced by 99.3%. Additionally, the leaching concentration of Ag decreased from 0.014 mg/L to 0.006 mg/L, while that of Ba decreased from 0.249 mg/L to 0.088 mg/L. Stainless SS was carbonated at 0.4 MPa and 300 °C for 120 min, and a thin calcite coating formed on the SS matrix surface. The leaching tests revealed that the chromium diffusion rates of fresh SS and CSS ranged from 1.32 × 10^−11^ min^−1^ to 2.12 × 10^−11^ min^−1^ and from 0.617 × 10^−11^ min^−1^ to 1.47 × 10^−11^ min^−1^, respectively. The inhibition of the diffusion rate by the calcite coating was approximately 30% to 53%. The diffusion activation energy of chromium was enhanced from 6.84 kJ/mol to 12.51 kJ/mol due to carbonation. The chromium leaching barrier of the calcite coating increased by 82.9%. This indicates that carbonation can effectively inhibit the leaching of chromium ions from stainless SS.

The carbonation of SS inhibits the leaching of heavy metal ions. However, some studies have also indicated that toxic vanadium is released into the solution as V(V) during the dissolution of dicalcium silicate in BOF slag and that some vanadium is incorporated into the newly formed C-S-H [114,115,116]. Vanadium may be leached in high-pH leachate during weathering. The converter SS released less than 0.1 mg/L of V. The concentrations of vanadium leached from the carbonated converter SS were 4.335 mg/L, 10.653 mg/L, and 18.831 mg/L when the carbonation conversions of the SS were 33.35%, 43.76%, and 58.95%, respectively [103]. This indicates that semidry carbonation promoted vanadium leaching and may have affected the cement hydration process.

### 4.7. Hydration Kinetics of CSS Cement

The addition of CSS promotes cement hydration, and the hydration rate of cement further accelerates with an increasing CSS dosage [78]. The CO_2_ uptake of CSS also affects the hydration process of cement. The low CO_2_ uptake of SS can accelerate cement hydration compared to that of uncarbonated SS cement at the same dosage, while the high CO_2_ uptake of SS delays cement hydration [102,103].

**Figure 11:**
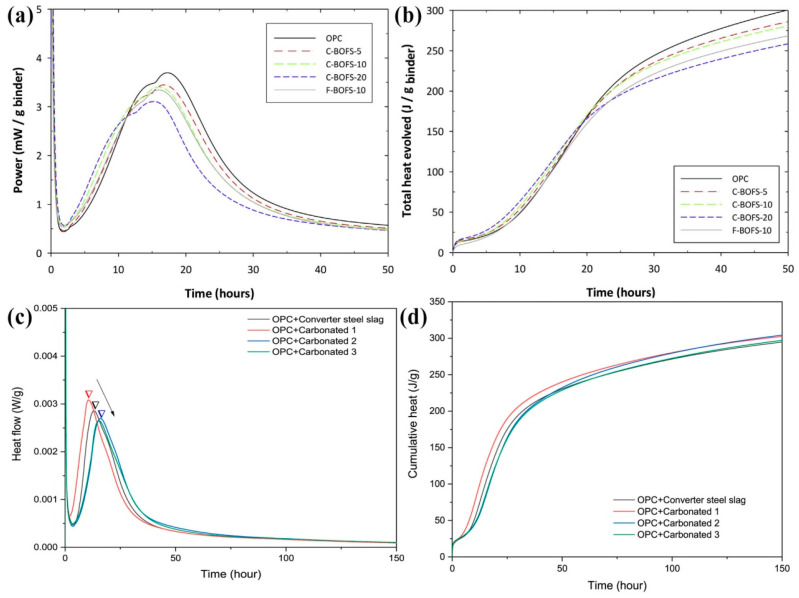
The heat evolution of the SS mixtures: (**a**) heat evolution; (**b**) total heat evolution [78]; (**c**) heat flow; (**d**) cumulative heat [103] (F-BOFS with a carbonation conversion of 6%; C-BOFS with a carbonation conversion of 60%; Carbonated 1 with a carbonation conversion of 33.35%; Carbonated 2 with a carbonation conversion of 43.76%; Carbonated 3 with the highest carbonation conversion of 58.95%).

The heat flow and cumulative heat of the CSS binder determined by isothermal calorimetry are shown in Figure 11. Figure 11a,b show the effect of different C-BOFS contents (5%, 10%, and 20%) on the heat of hydration. The shape of the heat evolution curve of C-BOFS was similar to that of OPC, showing two main hydration peaks due to calcium silicate and aluminate hydration [78]. The exothermic hydration process in the C-BOFS-10 paste was faster than that in the F-BOFS-10 paste and OPC. This is because the fine calcite powder could provide an additional surface for the nucleation and growth of hydrates. The hydration rate increased with an increasing C-BOFS content, as carbonate deposition provided more nucleation sites. Due to the effect of clinker dilution, the cumulative heat and maximum heat flow during 50 h decreased with an increasing C-BOFS content. The cumulative hydration heats of the OPC control, C-BOFS-5, C-BOFS-10, C-BOFS-20, and F-BOFS-10 pastes after 50 h were 300, 285, 280, 258, and 268 J/g, respectively.

Figure 11c,d show the effect of CSS with different carbonation conversions on the hydration of the SS–cement binder systems. The cumulative heat of the samples rapidly increased before the first 48 h, followed by a slow growth [103]. The cumulative heats of converter SS, carbonated 1, carbonated 2, and carbonated 3 after 150 h were 294.6 J/g, 302.3 J/g, 303.9 J/g, and 297.0 J/g, respectively. Compared with the addition of uncarbonated SS, the addition of CSS promoted cement clinker hydration. In addition, the carbonation conversion of CSS affected the cement binder hydration process. A low carbonation conversion of CSS can accelerate cement hydration, while a high carbonation conversion of CSS retards cement hydration. The incorporation of CSS introduces silica gel and calcium carbonate to the cement binder system. They act as additional nucleation sites and provide more sources of Ca and Si for C-S-H formation during hydration. However, more vanadium is leached with increasing carbonation conversion, which may lead to the retardation of cement hydration.

### 4.8. Microstructure of CSS Cement

**Figure 12:**
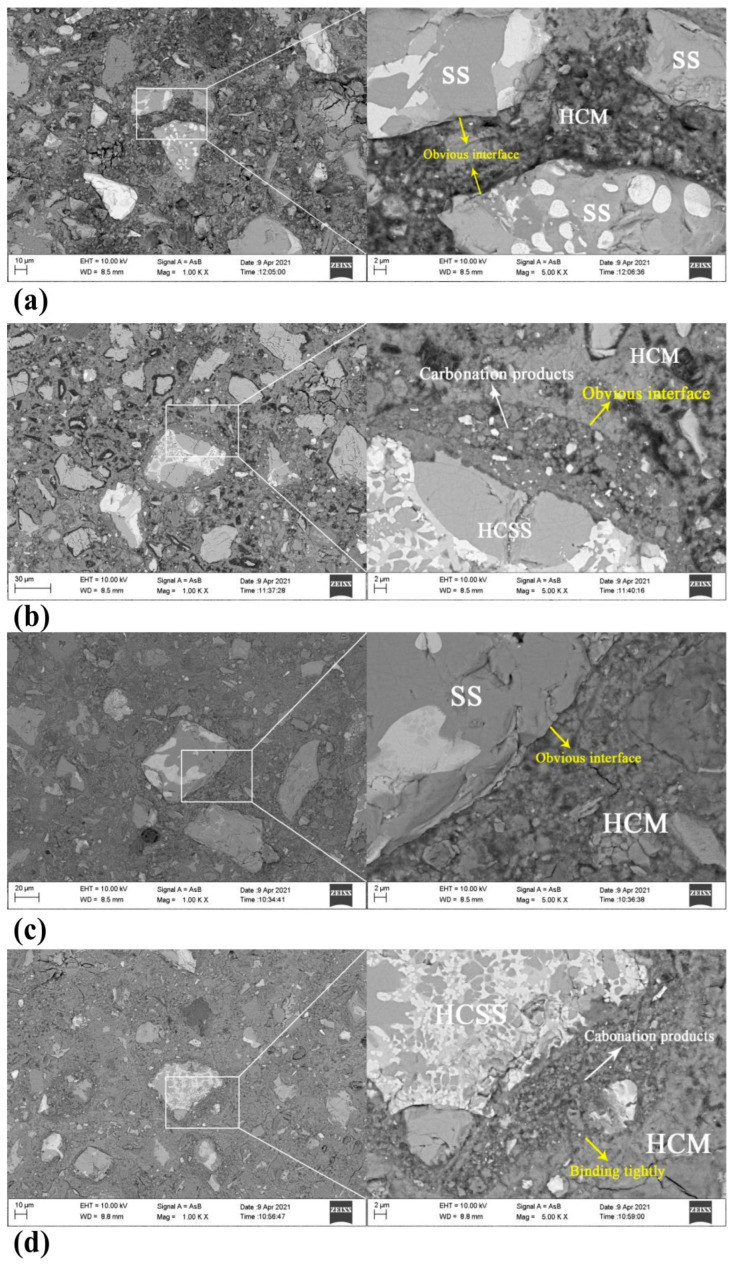
Typical BSEM images of cement pastes containing SS: (**a**) SS cement paste at 3 d; (**b**) HCSS cement paste at 3 d; (**c**) SS cement paste at 28 d; and (**d**) HCSS cement paste at 28 d (HCM: hydrated cement matrix) [73].

Compared with that of the SS paste, the total porosity of the CSS paste slightly increases, and the proportion of pores with sizes smaller than 20 nm increases significantly [73]. The higher the carbonation conversion, the greater the total porosity. This may be due to the increase in the amount of hydrates generated by the pozzolanic reaction of the carbonation products in the CSS. Moreover, the porous microstructure of CSS may also lead to an increase in porosity. BSEM images of the SS and HCSS cement pastes at 3 d and 28 d are shown in Figure 12. The presence of clear interfaces between the hydrated cement matrix and SS particles at both 3 d and 28 d indicates that uncarbonated SS is difficult to hydrate and provide hydration products to enhance the interfacial adhesion [73]. Some pores are found in the HCSS cement paste at 3 d compared to the SS cement paste, and the carbonation products of HCSS bond strongly with the hydrated cement matrix at 28 d, resulting in a dense microstructure. This may be due to the pozzolanic reaction of the amorphous SiO_2_ gel with Ca(OH)_2_, which enhances the strength of the HCSS cement paste.

## 5. Conclusions and Prospects

SS and CO_2_ emissions from the steel industry cause environmental damage and waste of resources. The accelerated carbonation of SS can reduce CO_2_ emissions and remove the contents of f-CaO and f-MgO in SS. Using CSS as an SCM in cement can achieve the large-scale utilization of SS while reducing CO_2_ emissions, which is significant for the low-carbon sustainable development of the iron and steel industry. In this paper, the main routes and mechanisms of the accelerated carbonation of SS are summarized. The physicochemical properties of CSS are reviewed, and the effects of carbonation on the mineral composition, physical properties, and CO_2_ uptake of SS are compared. In addition, the effects of using CSS as an SCM in cement on the mechanical properties, workability, volume stability, durability, environmental performance, hydration kinetics, and microstructure of the materials are analyzed and evaluated. The challenges and prospects for the application of CSS in the cement and concrete industry are summarized and discussed. The following conclusions are drawn.

The mineral phase and crystallization morphology of CSS are closely related to the mineral type of the SS and the carbonation conditions in which the SS mineral phase has a greater influence. The content of active calcium in SS determines the carbonation activity. Calcium in the form of f-CaO and portlandite has the highest susceptibility to carbonation. The carbonation activities of C_2_S and C_3_S are the next highest. γ-C_2_S has a greater carbonation reactivity than β-C_2_S. Calcium oxide minerals structurally bound to elements (Mg, Fe, or Al) exhibit low carbonation reactivity. Other minor oxides, such as FeO, calcium iron oxide, and magnbesioferrite, do not participate in the carbonation reaction. The carbonation products are near-amorphous C-S-H and calcium carbonate, the main form of which is calcite with traces of aragonite and magnesite.

The carbonation reaction of SS leads to a decrease in the average density, an increase in the specific surface area, a refinement of the pore structure, and the precipitation of different forms of calcium carbonate on the CSS surface. The higher the CO_2_ uptake of CSS, the lower its average density and the higher its specific surface area and total pore volume. Carbonation can increase the specific surface area of CSS by about 24–80%. The surface of uncarbonated SS is smooth and dense without crystalline precipitation. Rhombic or cubic calcite crystals of 0.5–3 μm in size, as well as needle-like aragonite, are attached to the surface of CSS after carbonation.

The CO_2_ uptake of CSS is 2–27 g/100 g SS, which is related to the carbonation time, temperature, CO_2_ concentration, L/S, gas flow rate, and particle size. The CO_2_ uptake is good when the stirring speed reaches 300–500 rpm and the L/S is 10.

The CSS properties, dosage, and CO_2_ uptake all affect the properties of CSS-based materials. The carbonation of SS can improve the mechanical properties of SS cement mortar, which can be attributed to both physical and chemical effects. The compressive strength of the CSS mortar first increases and then decreases with increasing CO_2_ uptake. Appropriate CO_2_ uptake can improve the hydration activity of SS, and excessive carbonation reduces the hydration activity. The excessive CO_2_ uptake of CSS may consume calcium silicate via hydration reactions, such as β-C_2_S and C_3_S, which adversely affects the early hydration activity of CSS and decreases the early strength of cementitious materials.

The workability of CSS cement paste is reduced and the setting time is prolonged as the particle size of the CSS decreases and the CO_2_ uptake increases. The setting time of CSS cement paste decreases with an increasing CSS dosage. The water demand for CSS paste increases with increasing dosage and CO_2_ uptake of CSS.

Carbonation significantly reduces the f-CaO, f-MgO, and Ca(OH)_2_ contents of SS, which improves the bulk stability and sulfate resistance of the CSS cement mortar. Meanwhile, SS carbonation inhibits the leaching of heavy metal ions from the solid matrix because a calcium carbonate layer forms on the CSS surface.

The addition of CSS promotes cement hydration, and the hydration rate of cement further accelerates with an increasing CSS dosage. The CO_2_ uptake of CSS also affects the hydration process of cement. The low CO_2_ uptake of SS can accelerate cement hydration compared to that of uncarbonated SS cement at the same dosage, while the high CO_2_ uptake of SS delays cement hydration.

Future research can be carried out based on the following four aspects:

(1) The accelerated carbonation of SS not only consumes f-CaO and f-MgO but also consumes C_3_S, C_2_S, and other minerals with hydration activity, which reduces the hydration activity of SS. It is necessary to comprehensively consider how to achieve a balance between the volume stability and hydration activity of SS.
(2) The mechanism of carbonation products on cement hydration needs to be studied. The carbonation conditions can be adjusted to obtain carbonation products with different forms and properties, which is conducive to improving the hydration activity of CSS.
(3) The effect of CSS on the durability of the cement, including the resistance to sulfate erosion, chloride ion penetration, and freeze-thaw resistance, should be further investigated.
(4) The environmental impact and energy consumption of CSS in cement should be assessed through a life cycle assessment to achieve sustainability.

## Figures and Tables

**Table 1:** Changes in the physicochemical properties of SS before and after accelerated carbonation.

Route	Type	Properties	Description	Reference
Direct dry carbonation	Physical	Particle size	In stainless SS: increase from 14.961 μm to 15.992 μm	[72]
		Particle surface	In stainless SS: decrease from 0.672 m^2^/cm^3^ to 0.650 m^2^/cm^3^	[72]
		Microstructure	In stainless SS: uncarbonated SS with smooth surface, granular calcite phase on CSS matrix	[72]
	Chemical	Mineralogy	In stainless SS: calcite, merwinite, and C_2_S phase exist in CSS	[72]
Direct wet carbonation (L/S < 0.2)	Physical	Blaine’s fineness	Increase from 337 m^2^/kg to 500 m^2^/kg (4.6 g CO_2_/100 g SS) and 560 m^2^/kg (13.2 g CO_2_/100 g SS)	[73]
		Specific surface area	Increase by 77.11% from 3.028 m^2^/g to 5.363 m^2^/g	[74]
			Increase from 2996 m^2^/kg to 4798 m^2^/kg (4.6 g CO_2_/100 g SS) and 16,169 m^2^/kg (13.2 g CO_2_/100 g SS)	[73]
		Total pore volume	Increase from 0.0123 cm^3^/g to 0.0242 cm^3^/g (4.6 g CO_2_/100 g SS) and 0.0419 cm^3^/g (13.2 g CO_2_/100 g SS)	[73]
		Pore size	Pores with sizes of 3–10 nm increase	[74]
		Microstructure	Uncarbonated SS has smooth and dense surface; some porous products formed on particle surface at 15 min of carbonation, abundant carbonation products covered particle at 240 min of carbonation	[73]
			Rough and porous product layer with thickness of 5–10 µm formed around SS particles; product layer consisted of large amount of needle-like carbonation products with diameter of 10–20 nm	[74]
	Chemical	Mineralogy	β-C_2_S: decrease from 21.7% to 18.0% (4.6 g CO_2_/100 g SS) and 9.3% (13.2 g CO_2_/100 g SS)	[73]
			γ-C_2_S: decrease from 9.3% to 8.6% (4.6 g CO_2_/100 g SS) and 1.9% (13.2 g CO_2_/100 g SS)	
			Calcite: increase from 0.6% to 10.9% (4.6 g CO_2_/100 g SS) and 28.0% (13.2 g CO^2^/100 g SS)	
			Nano-CaCO_3_ and amorphous SiO_2_ gel as main carbonation products	[74]
		Carbonation activity	In BOFS: portlandite (Ca(OH)_2_) shows high carbonation susceptibility	[75]
			In BOFS: srebroloskite, merwinite, and cuspidine mineral exhibit low carbonation susceptibility	
Direct wet carbonation (L/S > 5)	Physical	Particle size	In BOFS: decrease from 8.73 μm to 6.99 μm (carbonation conversion = 17%) and 5.23 μm (carbonation conversion = 48%)	[76]
		Specific gravity	In BOFS: decrease from 3.56 g/cm^3^ to 2.47–3.27 g/cm^3^	[77]
			In BOFS: decrease from 3.13 g/cm^3^ to 2.66 g/cm^3^	[78]
			In BOFS: decrease from 3.14 g/cm^3^ to 2.81 g/cm^3^ (carbonation conversion = 17%) and 2.71 g/cm^3^ (carbonation conversion = 48%)	[76]
		Specific surface area	In BOFS: increase from 1.73 m^2^/g to 7.22–9.84 m^2^/g	[77]
			In BOFS: increase from 5879 cm^2^/g to 7318 cm^2^/g (carbonation conversion = 17%) and 9642 cm^2^/g (carbonation conversion = 48%)	[76]
			In BOFS: increase from 672 m^2^/kg to 1020 m^2^/kg	[78]
			In EAFS: increase from 1.7 m^2^/g to 13.9 m^2^/g	[79]
		Microstructure	Calcite particles in form of nonuniform particles and carbonation layer morphology on pore and crack surface of SS	[80]
			In BOFS: “rhombohedral-like” or “cubic-like” structures of calcite with sizes of 0.5–3 μm, “needle-like” structures of aragonite, CaCO_3_ product with regular morphology as compact continuous coatings on SS surface	[77]
			Fresh BOFS without crystallized precipitates on smooth surface, rhombohedral calcite crystals with size of 1–3 μm attached on surface of carbonated BOFS	[76]
			In EAFS: spindle-shaped product layer covers particles	[79]
			In LFS: no significant change in particle shape or surface	
	Chemical	Mineralogy	In BOFS: pure calcite as carbonation product	[81]
			In EAFS: Ca_3_Mg(SiO_4_)_2_ as main CO_2_ binding component, calcite as main carbonation product; Mg compounds are not reactive toward CO_2_ at these mild conditions	[79]
			Consumption: C_2_S, C_3_S, CaO, and Ca(OH)_2_ Formation: calcite	[80]
		Free-CaO and Ca(OH)_2_	In BOFS: free-CaO (5.1%) and Ca(OH)_2_ (1.8%) were totally eliminated	[77]
			In BOFS: free-CaO (0.8%) and Ca(OH)_2_ (7.7%) were totally eliminated	[76]
			In BOFS: free-CaO (1.02%) and Ca(OH)_2_ (7.68%) were eliminated	[78]
			Content of free-CaO reduced	[80]

**Table 2:** Phase compositions of carbonated BOFS shown as wt. % [75].

Phase	BOFS	30 °C-1 bar-4 h	30 °C-5 bar-4 h	60 °C-1 bar-4 h	60 °C-5 bar-4 h
Larnite (β-C_2_S)	8.6	9.5	7.1	5.4	3.8
Portlandite (Ca(OH)_2_)	11	2.2	1.6	1.7	1.6
Calcite (CaCO_3_)	1.8	12.5	24.1	22.3	28.5
Srebrodolskite (Ca_2_Fe_2_O_5_)	21	16.5	19.8	17.9	17.3
FeO	1.7	1.2	2.4	2.5	2.0
Magnesioferrite (Fe_2_Mg_4_O_4_)	4.3	4.4	4.8	4.4	4.0
Ca,Fe oxide	2	2.1	2.2	1.8	1.4
P_2_O_5_	1	0.6	0.5	0.7	0.7
Cuspidine (Ca_4_Si_2_O_7_F_2_)	4	1.5	1.9	1.9	1.1
Merwinite (Ca_3_Mg(SiO_4_)_2_)	4.5	2.4	1.2	2.0	1.6
Amorphous	32.7	38.0	22.5	26.9	26.2

**Table 3:** The effect of direct carbonation on the CO_2_ uptake of SS powder.

Carbonation	SS Type	Particle Size (µm)	Temperature (°C)	L/S (mL/g)	CO_2_ Concentration (%)	CO_2_ Pressure (MPa)	CO_2_ Flow Rate (L/min)	Time (min)	CO_2_ Uptake (g/100 g SS)	Reference
Dry	EAFS + AODS	48–75	300	-	99.99	0.4	-	120	3.14	[72]
Wet	SS	3.028 m^2^/g	20 ± 2	0.08	99.9	0.25	-	30	6.14	[74]
Wet	SS	<80	20 ± 2	0.08	99.9	0.25	-	15	4.6	[73]
Wet	SS	<80	20 ± 2	0.08	99.9	0.25	-	240	13.2	[73]
Wet	BOFS	<44	60	10	-	0.1013	0.1	60	27.2	[81]
Wet	BOFS	62	25	20	28.2	0.1013	4.7	-	7.02	[77]
Wet	BOFS	12.65	60	20	99	-	3	180	21.5	[78]
Wet	EAFS	<100	Normal	10	15	0.1013	0.83	65	8.7	[79]
Wet	EAFS	<100	Normal	10	15	0.1013	0.83	65	1.9	[79]
Wet	LFS	24	Normal	10	15	0.1013	0.83	65	4.6	[79]

**Table 5:** Fresh properties and setting times of OPC and CSS cement pastes.

Items	CO_2_ Uptake (g/100 g SS)	Particle Size (µm)	Workability (mm)	Water Demand (*w*/*w*%)	Setting Time (min)		Reference
					Initial	Final	
OPC	-	14.9	122.0 ± 1.9	25.8 ± 0.2	211 ± 6	251 ± 4	[99]
CS1-10	3.79	120.8	123.0 ± 2.3	25.9 ± 0.1	270 ± 5	310 ± 4	
CS2-10	6.92	69.8	117.5 ± 0.9	26.2 ± 0.1	276 ± 4	316 ± 5	
CS3-10	8.31	35.6	115.2 ± 1.3	27.3 ± 0.3	302 ± 6	348 ± 5	
CS4-10	8.85	19.1	113.9 ± 2.5	29.0 ± 0.2	309 ± 4	355 ± 5	
OPC	-	17.88	-	25.1 ± 0.03	150 ± 3	261 ± 3	[78]
F-BOFS-10	2.03	11.44	-	25.1 ± 0.01	150 ± 4	265 ± 5	
C-BOFS-10	20.3	9.67	-	26.9 ± 0.08	145 ± 2	260 ± 1	
OPC	-	-	-	26.9	275	340	[110]
SS-5	0.4	-	-	27.69	255	270	
SS-10	0.4	-	-	28.46	124	270	
SS-15	0.4	-	-	29.23	215	280	
SS-20	0.4	-	-	30.00	96	305	
CSS-5	15.1	-	-	31.5	262	325	
CSS-10	15.1	-	-	32.3	210	315	
CSS-15	15.1	-	-	33.1	230	285	
CSS-20	15.1	-	-	33.8	205	185	
Limits	-	-	-	-	>45	<375	

## Data Availability

The original contributions presented in this study are included in this article; further inquiries can be directed to the corresponding author.
